# Remote Ischemic Preconditioning to Prevent Acute Kidney Injury After Cardiac Surgery: A Meta-Analysis of Randomized Controlled Trials

**DOI:** 10.3389/fcvm.2021.601470

**Published:** 2021-03-18

**Authors:** Zigang Liu, Yongmei Zhao, Ming Lei, Guancong Zhao, Dongcheng Li, Rong Sun, Xian Liu

**Affiliations:** ^1^Department of Thoracic and Cardiovascular Surgery, Shenzhen Baoan Women's and Children's Hospital, Jinan University, Guangzhou, China; ^2^Center for Cardiac Intensive Care, Beijing Anzhen Hospital, Beijing, China

**Keywords:** remote ischemic preconditioning, acute kidney injury, cardiac surgery, off-pump, meta-analysis

## Abstract

**Objective:** Randomized controlled trials (RCTs) evaluating the influence of remote ischemic preconditioning (RIPC) on acute kidney injury (AKI) after cardiac surgery showed inconsistent results. We performed a meta-analysis to evaluate the efficacy of RIPC on AKI after cardiac surgery.

**Methods:** Relevant studies were obtained by search of PubMed, Embase, and Cochrane's Library databases. A random-effect model was used to pool the results. Meta-regression and subgroup analyses were used to determine the source of heterogeneity.

**Results:** Twenty-two RCTs with 5,389 patients who received cardiac surgery −2,702 patients in the RIPC group and 2,687 patients in the control group—were included. Moderate heterogeneity was detected (*p* for Cochrane's *Q* test = 0.03, *I*^2^ = 40%). Pooled results showed that RIPC significantly reduced the incidence of AKI compared with control [odds ratio (OR): 0.76, 95% confidence intervals (CI): 0.61–0.94, *p* = 0.01]. Results limited to on-pump surgery (OR: 0.78, 95% CI: 0.64–0.95, *p* = 0.01) or studies with acute RIPC (OR: 0.78, 95% CI: 0.63–0.97, *p* = 0.03) showed consistent results. Meta-regression and subgroup analyses indicated that study characteristics, including study design, country, age, gender, diabetic status, surgery type, use of propofol or volatile anesthetics, cross-clamp time, RIPC protocol, definition of AKI, and sample size did not significantly affect the outcome of AKI. Results of stratified analysis showed that RIPC significantly reduced the risk of mild-to-moderate AKI that did not require renal replacement therapy (RRT, OR: 0.76, 95% CI: 0.60–0.96, *p* = 0.02) but did not significantly reduce the risk of severe AKI that required RRT in patients after cardiac surgery (OR: 0.73, 95% CI: 0.50–1.07, *p* = 0.11).

**Conclusions:** Current evidence supports RIPC as an effective strategy to prevent AKI after cardiac surgery, which seems to be mainly driven by the reduced mild-to-moderate AKI events that did not require RRT. Efforts are needed to determine the influences of patient characteristics, procedure, perioperative drugs, and RIPC protocol on the outcome.

## Introduction

Acute kidney injury (AKI) is common for patients after cardiac surgery, particularly for patients who receive cardiac surgery with complex procedures, such as on-pump surgery, concomitant coronary artery bypass graft (CABG) with valvular surgery, longer aortic cross-clamp time during cardiopulmonary bypass, and surgeries of open total aortic arch replacement (1–3). Previous studies showed that patients with postoperative AKI, even of mild degree, had worse clinical outcome after cardiac surgery (4). Currently, multiple criteria have been applied to define AKI after cardiac surgery, while consensus regarding the optimal definition of AKI remains lacking (5). Among which, the Acute Kidney Injury Network (AKIN) and the Risk, Injury, Failure, Loss, End Stage Kidney Disease (RIFLE) have been the mostly used criteria to define AKI, while the Kidney Disease: Improving Global Outcomes (KDIGO) criteria for AKI staging has been shown to demonstrate greater sensitivity to detect AKI and to predict associated in-hospital mortality, than do the RIFLE or AKIN criteria (5). Since treatment options for postoperative AKI remain limited, identification of strategies to prevent the incidence of AKI after cardiac surgery are clinically important (6). Remote ischemic preconditioning (RIPC), which is known as a strategy that protects the target organ by inducing brief episodes of ischemia and reperfusion in distant tissue, has been suggested to be effective for preventing AKI (7–9). However, randomized clinical trials (RCTs) evaluating the influence of RIPC on AKI after cardiac surgery showed inconsistent results (10–31). Since most of these studies were of limited sample size, the individual studies may have been statistically insufficient to detect a significant influence of RIPC on postoperative AKI. Furthermore, several previous meta-analyses that pooled studies published through 2016 showed that RIPC was not effective at preventing AKI in patients after on-pump cardiac surgery (32–34). Although, these results were also inconsistent (35). Many RCTs have been published since 2016 (22, 24–31), warranting an updated meta-analysis. In addition, the potential impacts of study and patient features on the efficacy of RIPC on postoperative AKI in these patients were rarely analyzed. Therefore, we performed an updated meta-analysis with comprehensive subgroup analyses to investigate the role of RIPC to prevent AKI after cardiac surgery.

## Methods

The Preferred Reporting Items for Systematic Reviews and Meta-Analyses (PRISMA) statement (36) and the Cochrane Handbook guidelines (37) were followed during the designing and implementation of the study.

### Search Strategy

PubMed, Embase, and the Cochrane Library (Cochrane Center Register of Controlled Trials) databases were searched for relevant studies with a combined strategy of: [1] “ischemic preconditioning” OR “remote ischemic preconditioning” OR “RIPC”; [2] “cardiac surgery” OR “coronary artery bypass” OR “surgical coronary revascularization” OR “valve surgery” OR “valve replacement”; and [3] “random” OR “randomized” OR “randomized” OR “randomly.” Only clinical studies were considered. The references of related reviews and original articles were also searched as a complementation. The latest database search was conducted on 5th May 2020.

### Study Selection

Inclusion criteria were as follows: [1] peer-reviewed articles in English or Chinese; [2] designed as parallel-group RCTs; [3] included adult patients scheduled for open heart surgery who were randomly allocated to a RIPC treatment group or a control group; and [4] reported the incidence of AKI in the perioperative periods. Reviews, studies including children or neonates, preclinical studies, observational studies, and repeated reports were excluded.

### Data Extraction and Quality Assessment

Study search, data extraction, and quality evaluation were achieved by two independent authors. If disagreement occurred, it was resolved by consensus between the two authors. We extracted data regarding study information (first author, publication year, and study country), study design (blind or open-label), patient information (number of participants, mean age, gender, diabetic status, and patients with severe cardiac systolic dysfunction), surgery type, perioperative anesthetics (use of propofol or volatile anesthetics, and cross-clamp time), RIPC protocol, and definition of AKI. Quality evaluation was achieved using the Cochrane's Risk of Bias Tool (37) according to the following aspects: [1] random sequence generation; [2] allocation concealment; [3] blinding of participants and personnel; [4] blinding of outcome assessors; [5] incomplete outcome data; [6] selective outcome reporting; and [7] other potential bias.

### Statistical Analysis

Incidence of AKI in each arm was evaluated via odds ratio (OR) and its 95% confidence intervals (CIs). We used the Cochrane's *Q* test to detect the heterogeneity, and significant heterogeneity was suggested if *p* < 0.10 (38). The *I*^2^ statistic was also calculated, and an *I*^2^ > 50% reflected significant heterogeneity. Pooled analyses were calculated using a random-effect model because this method incorporates the influence of potential heterogeneity and retrieves a more generalized result (37). Stratified analyses comparing the results in complex and simple cardiac surgeries were also performed. We defined complex surgeries as double-valve or triple-valve surgery, mitral valve surgery, coronary artery bypass graft (CABG) plus valve(s), open total aortic arch replacement, or any “redo” operation. Accordingly, CABG or single-valve surgeries were defined as simple surgeries. Predefined meta-regression analyses, sensitivity analyses, and subgroup analyses were performed to explore the potential influences of study characteristics on the outcome. These characteristics included study design, country, age, gender, diabetic status, surgery type, use of propofol, or volatile anesthetics, cross-clamp time, RIPC protocol, definition of AKI, and sample size of the RCT (39). For continuous variables, medians were used for cut-off. To evaluate the influence of RIPC on the AKI events with different severity, we defined patients with mild-to-moderate AKI as those that did not require renal replacement therapy (RRT) and those with severe AKI as patients that required RRT. Stratified analysis was performed accordingly. Publication bias was evaluated by visual inspection of funnel plots, and the Egger's regression asymmetry test (40). *p* < 0.05 were considered statistically significant. The RevMan (Version 5.1; Cochrane, Oxford, UK) and Stata software (Version 12.0; Stata, College Station, TX) were applied for statistical analyses.

## Results

### Search Results

**Figure 1:**
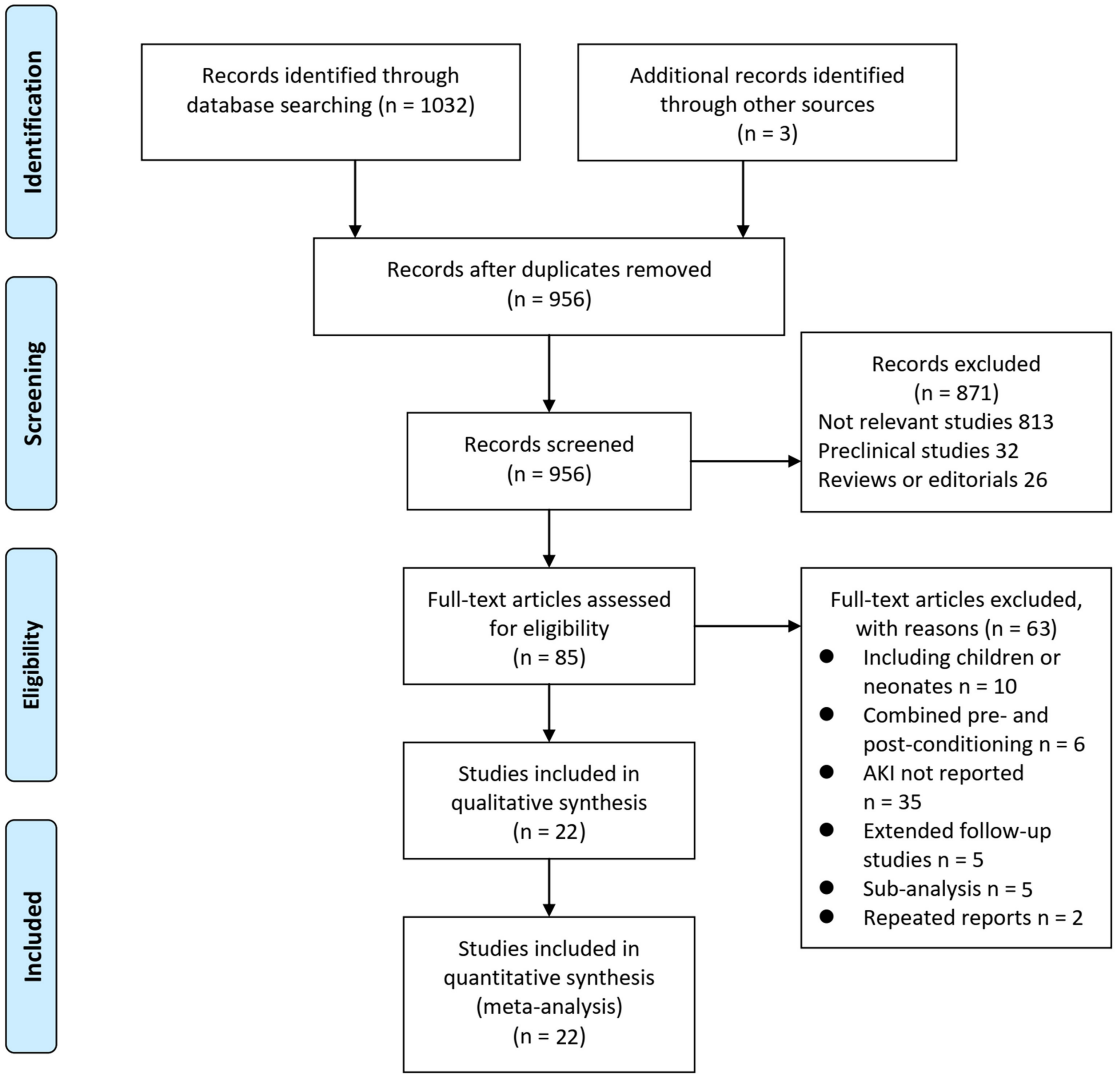
Flowchart of literature search.

In summary, 1,035 articles were obtained through the database search. After exclusion of duplicate studies, 956 articles were screened. Among them, 871 articles were subsequently excluded based on titles and abstracts primarily because these studies were irrelevant. Among the 85 potentially relevant articles, 63 were further excluded via full-text review based on reasons listed in Figure 1. Finally, 22 RCTs (10–31) were included.

### Study Characteristics

Table 1 shows the characteristics of the included studies. Overall, 22 RCTs (10–31) with 5,389 patients who received cardiac surgery −2,702 patients in the RIPC group and 2,687 patients in the control group—were included. These studies were published between 2010 and 2020. Patients with normal kidney function at baseline were included except one study that included patients with chronic kidney disease (CKD) who were scheduled for cardiac surgery (17). With regard to the surgery procedures, 20 studies included patients who received on-pump surgeries (10–28, 30), while the remaining two studies included patients who received off-pump surgeries (29, 31). Proportions of diabetic patients varied among the included studies, and patients with severe cardiac systolic function (left ventricular ejection fraction <30%) were rarely included. The use of propofol and volatile anesthetics varied among the patients of the included studies. RIPC was performed after anesthesia induction and before CPB in most of the included studies (acute RIPC), except in one study in which RIPC was performed 24–48 h before the surgery (chronic RIPC) (25). The protocol of RIPC included 3–4 cycles of upper or lower limb ischemia (5–10 min blood pressure cuff inflation to a pressure of 200 mmHg or at least a pressure that was 20 mmHg higher than the systolic arterial pressure), followed by 5–10 min reperfusion (with the cuff deflated). Uninflated cuffs were used on patients in the control group. Most of the studies used the AKIN definition of AKI, while the RIFLE (13, 19, 28) and the KDIGO (18, 20, 30, 31) definitions were used in three and four studies, respectively.

**Table 1:** Characteristics of the included studies.

**Study**	**Country**	**Design**	**Surgical procedure**	**No. of patients**	**Mean age (years)**	**Male (%)**	**DM (%)**	**Baseline eGFR (ml/min)**	**LVEF <30% (%)**	**Propofol used**	**Volatile anesthetics**	**Cross-clamp time (min)**	**Protocols of RIPC**	**Control**	**Definitions of AKI**
Venugopal et al. (10)	UK	R, SB	On-pump CABG with or without AVR	78	65.1	82.1	0	NR	2.6	Partial	61.5	52	UL, 200 mmHg, 5 min × 3, after anesthesia induction and before CPB	Uninflated cuff	AKIN
Choi et al. (2011)	South Korea	R, DB	On-pump complex valvular heart surgery	76	68.5	39.5	6.6	78.5	0	None	100	103	LL, 250 mmHg, 10 min × 3, after anesthesia induction and before CPB	Uninflated cuff	AKIN
Zimmerman et al. (2011)	USA	R, SB	On-pump CABG with or without AVR	118	63.5	68.5	38.1	NR	10.2	NR	100	71	LL, 200 mmHg, 5 min × 3, after anesthesia induction and before CPB	Uninflated cuff	AKIN
Young et al. (13)	New Zealand	R, DB	On-pump complex heart surgery	96	65.0	62.5	5.2	NR	4.2	All	100	111	UL, 200 mmHg, 5 min × 3, after anesthesia induction and before CPB	Uninflated cuff	RIFLE
Meybohm et al. (14)	Germany	R, DB	On-pump heart surgery	180	69.0	81.2	21.1	NR	0	All	0	80	UL, 200 mmHg, 5 min × 4, after anesthesia induction and before CPB	Uninflated cuff	AKIN
Wang et al. (15)	China	R, DB	On-pump valvular heart surgery	31	49.4	33.1	0	NR	NR	NR	NR	54	LL, 600 mmHg, 5 min × 3, after anesthesia induction and before CPB	Uninflated cuff	AKIN
Candilio et al. (16)	UK	R, DB	On-pump CABG with or without valvular surgery	178	65.5	78.0	29.5	NR	4.5	All	100	63	UL and LL, 200 mmHg, 5 min × 2, after anesthesia induction and before CPB	Uninflated cuff	AKIN
Zarbock et al. (20)	Germany	R, DB	On-pump heart surgery	240	70.4	62.9	37.5	56.6	15	None	100	82	UL, 200 mmHg, 5 min × 3, after anesthesia induction and before CPB	Uninflated cuff	KDIGO
Gallagher et al. (17)	UK	R, SB	CKD patients that received on-pump CABG with or without AVR	86	70.8	80.2	64.0	51	10.4	NR	87.2	62	UL, SBP + 50 mmHg, 5 min × 3, after anesthesia induction and before CPB	Uninflated cuff	AKIN
Meybohm et al. (19)	Germany	R, DB	On-pump heart surgery	1,385	65.9	74.2	24.9	NR	0	All	0	77	UL, 200 mmHg, 5 min × 4, after anesthesia induction and before CPB	Uninflated cuff	RIFLE
Hausenloy et al. (18)	UK	R, DB	On-pump CABG with or without valvular surgery	1,612	76.2	71.6	25.9	NR	NR	All	42.1	70	UL, 200 mmHg, 5 min × 4, after anesthesia induction and before CPB	Uninflated cuff	KDIGO
Walsh et al. (24)	Canada, USA, India, and China	R, DB	On-pump heart surgery	258	72.2	58.5	30.6	NR	NR	Partial	84.4	99	LL, 300 mmHg, 5 min × 3, after anesthesia induction and before CPB	Uninflated cuff	AKIN
Pinaud et al. (23)	France	R, SB	On pump AVR with or without CABG	99	73.4	51.5	14.1	92.4	0	All	100	57	UL, 200 mmHg, 5 min × 3, after anesthesia induction and before CPB	Uninflated cuff	AKIN
Hu et al. (21)	China	R, DB	On-pump valvular heart surgery	201	47.1	37.8	0	NR	NR	All	100	59	LL, 600 mmHg, 5 min × 3, after anesthesia induction and before CPB	Uninflated cuff	AKIN
Nouraei et al. (22)	Iran	R, DB	On-pump CABG without valvular surgery	99	60.3	70.7	46.5	NR	0	Partial	38.5	41	LL, SBP + 20 mmHg, 5 min × 3, after anesthesia induction and before CPB	Uninflated cuff	AKIN
Song et al. (26)	South Korea	R, DB	On-pump AVR due to aortic stenosis	72	66.5	50.0	0	NR	0	None	100	59	UL, 300 mmHg, 5 min × 3, after anesthesia induction and before CPB	Uninflated cuff	AKIN
Kim et al. (25)	South Korea	R, DB	On-pump heart surgery	160	62.3	53.1	0	NR	0	All	0	147	UL, 200 mmHg, 5 min × 4, 24~48 h before surgery	Uninflated cuff	AKIN
Bagheri et al. (27)	Iran	R, DB	On-pump CABG without valvular surgery	180	63.6	57.6	35.0	NR	8	Partial	8.5	29	UL, 200 mmHg, 5 min × 3, after anesthesia induction and before CPB	Uninflated cuff	AKIN
Gasparovic et al. (28)	USA	R, DB	On-pump CABG without valvular surgery	66	62.0	82.0	36.0	NR	0	None	100	56	UL, 200 mmHg, 5 min × 3, after anesthesia induction and before CPB	Uninflated cuff	RIFLE
Wang et al. (29)	China	R, DB	Off-pump CABG without valvular surgery	65	60.5	73.5	NR	NR	0	None	100	NA	UL, SBP + 40 mmHg, 5 min × 4, after anesthesia induction and before CPB	Uninflated cuff	AKIN
Zhou et al. (35)	China	R, DB	Open TAAR under CPB with or without CABG	130	46.6	54	6.2	NR	NR	Partial	32.3	96	UL, SBP + 50 mmHg, 5 min × 4, after anesthesia induction and before CPB	Uninflated cuff	KDIGO
Stokfisz et al. (31)	Poland	R, DB	Off-pump CABG without valvular surgery	28	66.0	65.5	46.5	NR	NR	All	0	NA	UL, 200 mmHg, 5 min × 3, after anesthesia induction and before CPB	Uninflated cuff	KDIGO

*DM, diabetes mellitus; eGFR, estimated glomerular filtrating rate; LVEF, left ventricular ejection fraction; RIPC, remote ischemic preconditioning; AKI, acute kidney injury; UK, United Kingdom; USA, United States of America; R, randomized; DB, double-blinded; SB, single-blinded; AVR, aortic valvular replacement; TAAR, total aortic arch replacement; CKD, chronic kidney disease; CABG, coronary artery bypass graft; NR, not reported; NA, not applicable; UL, upper limb; LL, lower limb; SBP, systolic blood pressure; CPB, cardiopulmonary bypass; AKIN, Acute Kidney Injury Network; RIFLE, Risk, Injury, Failure, Loss, End-Stage Kidney Disease; KDIGO, Kidney Disease: Improving Global Outcomes*.

### Data Quality

Table 2 shows the details of study quality evaluation. Most of the included RCTs were double blinded except for four studies, which were single blinded (10, 12, 17, 23). Methods of random sequence generation were reported in 19 studies, and information of allocation concealment was reported in 14 studies. The overall quality score varied between 4 and 7, which suggested a generally good study quality.

**Table 2:** Details of study quality evaluation using the Cochrane's risk-of-bias tool.

**Study**	**Random sequence generation**	**Allocation concealment**	**Blinding of participants**	**Blinding of outcome assessment**	**Incomplete outcome data addressed**	**Selective reporting**	**Other sources of bias**	**Total**
Venugopal et sl. (10)	Low	Unclear	Low	High	Low	Low	High	4
Choi et al. (11)	Low	Unclear	Low	Low	Low	Low	Low	6
Zimmerman et al. (12)	Low	Low	Low	High	Low	Low	Unclear	5
Young et al. (13)	Low	Low	Low	Low	Low	Low	Low	7
Meybohm et al. (14)	Unclear	Low	Low	Low	Low	Low	Unclear	5
Wang et al. (15)	Low	Unclear	Low	Low	Low	Low	High	5
Candilio et al. (16)	Low	Low	Low	Low	Low	Low	Unclear	6
Zarbock et al. (20)	Low	Low	Low	Low	Low	Low	Low	7
Gallagher et al. (17)	Unclear	Unclear	Low	High	Low	Low	Low	4
Meybohm et al. (19)	Low	Low	Low	Low	Low	Low	Low	7
Hausenloy et al. (18)	Low	Low	Low	Low	Low	Low	Low	7
Walsh et al. (24)	Low	Low	Low	Low	Low	Low	Unclear	6
Pinaud et al. (23)	Low	Unclear	Low	High	Low	Low	Unclear	4
Hu et al. (7)	Unclear	Unclear	Low	Low	Low	Low	Unclear	4
Nouraei et al. (22)	Low	Low	Low	Low	Low	Low	Unclear	6
Song et al. (26)	Low	Low	Low	Low	Low	Low	Unclear	6
Kim et al. (25)	Low	Low	Low	Low	Low	Low	Unclear	6
Bagheri et al. (27)	Low	Low	Low	Low	Low	Low	Unclear	6
Gasparovic et al. (28)	Low	Low	Low	Low	Low	Low	Unclear	6
Wang et al. (29)	Low	Unclear	Low	Low	Low	Low	Unclear	5
Zhou et al. (30)	Low	Low	Low	Low	Low	Low	Low	7
Stokfisz et al. (31)	Low	Unclear	Low	Low	Low	Low	Unclear	5

### Meta-Analysis Results

**Figure 2:**
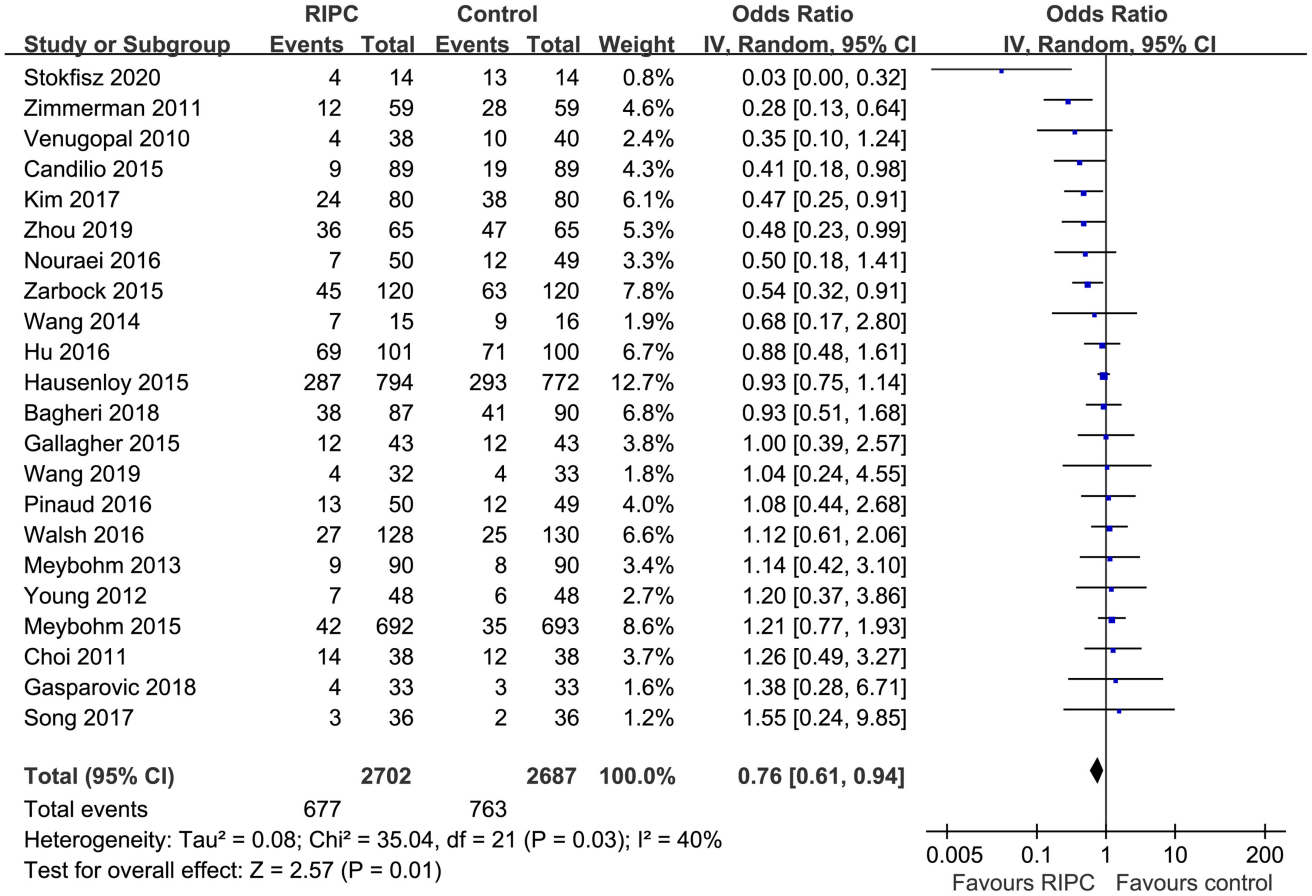
Forest plots for the meta-analysis comparing the efficacy of RIPC and control on AKI after cardiac surgery.

**Figure 3:**
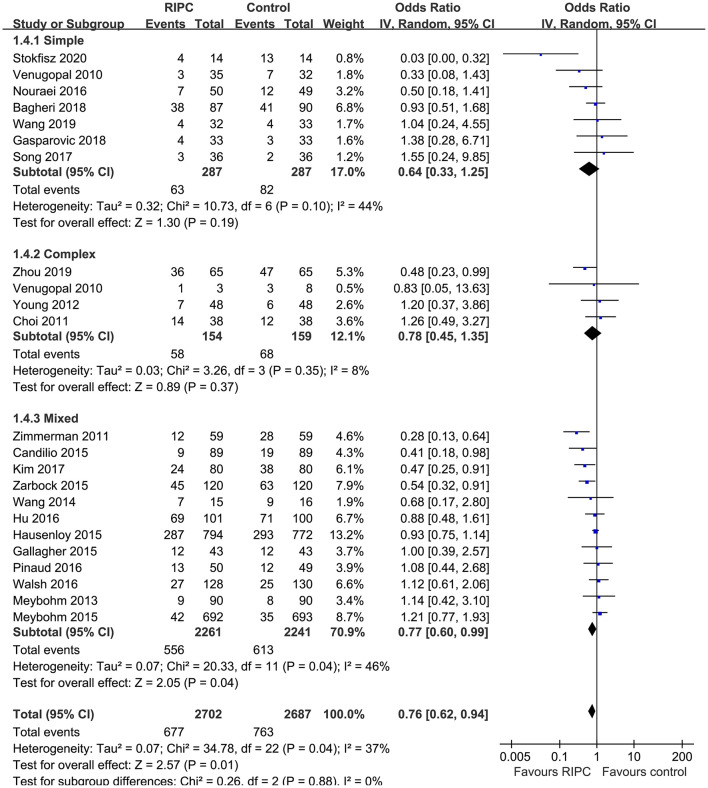
Stratified analyses according to the complexity of the surgery.

**Figure 4:**
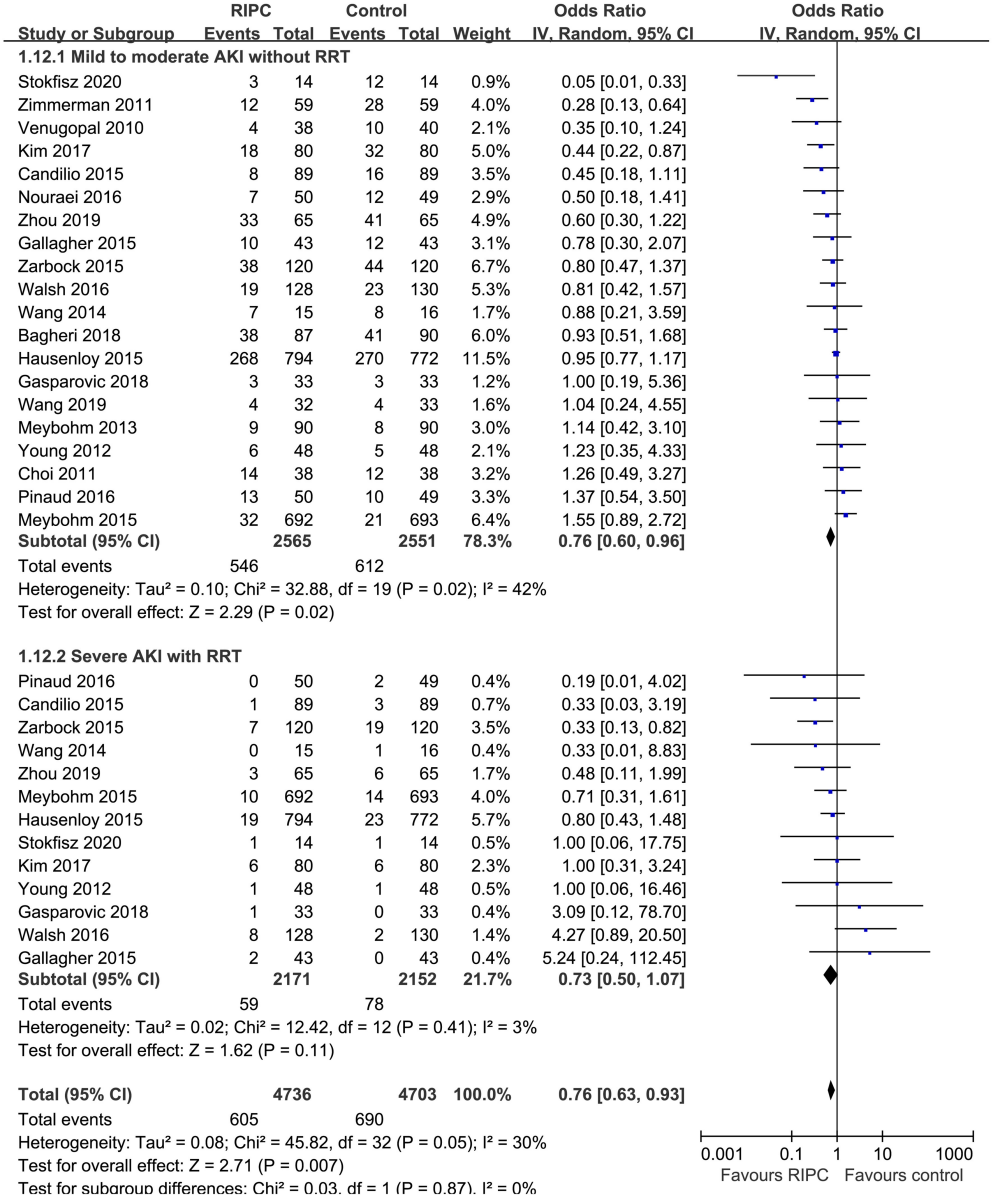
Stratified analyses according to the severity of AKI events.

Moderate heterogeneity was detected (*p* for Cochrane's *Q* test = 0.03, *I*^2^ = 40%) among the included RCTs. Pooled results with a random-effect model showed that RIPC significantly reduced the incidence of AKI after cardiac surgery compared with the control (OR: 0.76, 95% CI: 0.61–0.94, *p* = 0.01; Figure 2). Stratified analyses indicated that the effect of RIPC on postoperative AKI was not significantly different in studies with simple, complex, or mixed procedures (*p* for subgroup difference = 0.88, Figure 3). Results of the univariate meta-regression analyses suggested that study sample size, age, gender, diabetic status, volatile anesthetic use, and cross-clamp time in CPB did not significantly affect the outcome (*p* all >0.05; Table 3). Moreover, sensitivity analyses confirmed a potential preventative efficacy of RIPC on postoperative AKI in patients with normal renal function at baseline (21 studies, OR: 0.75, 95% CI: 0.60–0.93, *p* = 0.01), in studies with on-pump surgery only (20 studies, OR: 0.78, 95% CI: 0.64–0.95, *p* = 0.01), and in studies with acute RIPC (21 studies, OR: 0.78, 95% CI: 0.63–0.97, *p* = 0.03; Table 4). Further subgroup analyses indicated that study characteristics, including study design, country, age, gender, diabetic status, surgery type, use of propofol or volatile anesthetics, cross-clamp time, RIPC protocol, definition of AKI, or sample size of the RCT did not significantly affect AKI outcome (*p* for subgroup difference all >0.10; Table 4). Stratified analysis according to the severity of AKI showed that RIPC significantly reduced the risk of mild-to-moderate AKI that did not require RRT (OR: 0.76, 95% CI: 0.60–0.96, *p* = 0.02) but did not significantly reduce the risk of severe AKI that required RRT in patients after cardiac surgery (OR: 0.73, 95% CI: 0.50–1.07, *p* = 0.11; Figure 4).

**Table 3:** Results of univariate meta-regression analysis.

**Study characteristics**	**OR for the incidence of AKI after cardiac surgery**		
	**Coefficient**	**95% CI**	***p***
Number of subjects	0.13	−0.07 to 0.33	0.19
Mean age (years)	0.09	−0.07 to 0.25	0.27
Male (%)	0.02	−0.10 to 0.14	0.72
DM (%)	−0.02	−1.12 to 1.08	0.97
Volatile anesthetic use (%)	−0.12	−0.53 to 0.20	0.55
Cross-clamp time (min)	−0.17	−0.82 to 0.48	0.59

**Table 4:** Results of sensitivity and subgroup analyses.

**Study characteristics**	**Datasets number**	**OR (95% CI)**	***I*^**2**^**	***p* for subgroup effect**	***p* for subgroup difference**
Baseline renal function					
Only patients without CKD	21	0.75 [0.60, 0.93]	43%	0.01	–
Surgery characteristics					
Only studies of on-pump surgery	20	0.78 [0.64, 0.95]	31%	0.01	–
Timing of RIPC					
Only studies with acute RIPC	21	0.78 [0.63, 0.97]	38%	0.03	–
Study origin					
Asian	9	0.73 [0.55, 0.96]	0%	0.02	
Non-Asian	12	0.72 [0.51, 1.00]	59%	0.05	0.94
Study design					
DB	18	0.80 [0.64, 0.99]	35%	0.04	
SB	4	0.58 [0.28, 1.19]	55%	0.14	0.41
Surgery type					
CABG ± valvular surgery	10	0.66 [0.46, 0.96]	49%	0.03	
Valvular surgery ± CABG	5	0.99 [0.66, 1.50]	0%	0.96	
Any cardiac surgery	7	0.78 [0.55, 1.12]	50%	0.18	0.36
Propofol use					
All patients	10	0.72 [0.50, 1.02]	63%	0.06	
Partial patients	5	0.71 [0.47, 1.08]	29%	0.11	
None patients	5	0.75 [0.50, 1.12]	0%	0.16	0.99
Volatile anesthetics use					
All patients	10	0.72 [0.51, 1.02]	28%	0.06	
Partial patients	6	0.85 [0.68, 1.06]	12%	0.15	
None patients	4	0.61 [0.25, 1.49]	78%	0.28	0.62
Cross-clamp time (min)					
≤ 70	10	0.79 [0.59, 1.05]	0%	0.10	
>70	10	0.77 [0.58, 1.03]	56%	0.07	0.92
RIPC protocol					
Upper limb	16	0.76 [0.59, 0.98]	41%	0.03	
Lower limb	6	0.73 [0.46, 1.16]	46%	0.19	0.87
Definition of AKI					
AKIN	15	0.74 [0.58, 0.96]	19%	0.02	
RIFLE	3	1.22 [0.81, 1.85]	0%	0.34	
KDIGO	4	0.53 [0.28, 1.01]	78%	0.05	0.15
Sample size of RCT					
≤ 100	11	0.81 [0.53, 1.24]	22%	0.34	
>100	11	0.73 [0.57, 0.95]	55%	0.22	0.68

*CKD, chronic kidney disease; RIPC, remote ischemic preconditioning; AKI, acute kidney injury; DB, double-blinded; SB, single-blinded; CABG, coronary artery bypass graft; UL, upper limb; LL, lower limb; AKIN, Acute Kidney Injury Network; RIFLE, Acute Dialysis Quality Initiative; Kidney Disease Improving Global Outcomes*.

### Publication Bias

**Figure 5:**
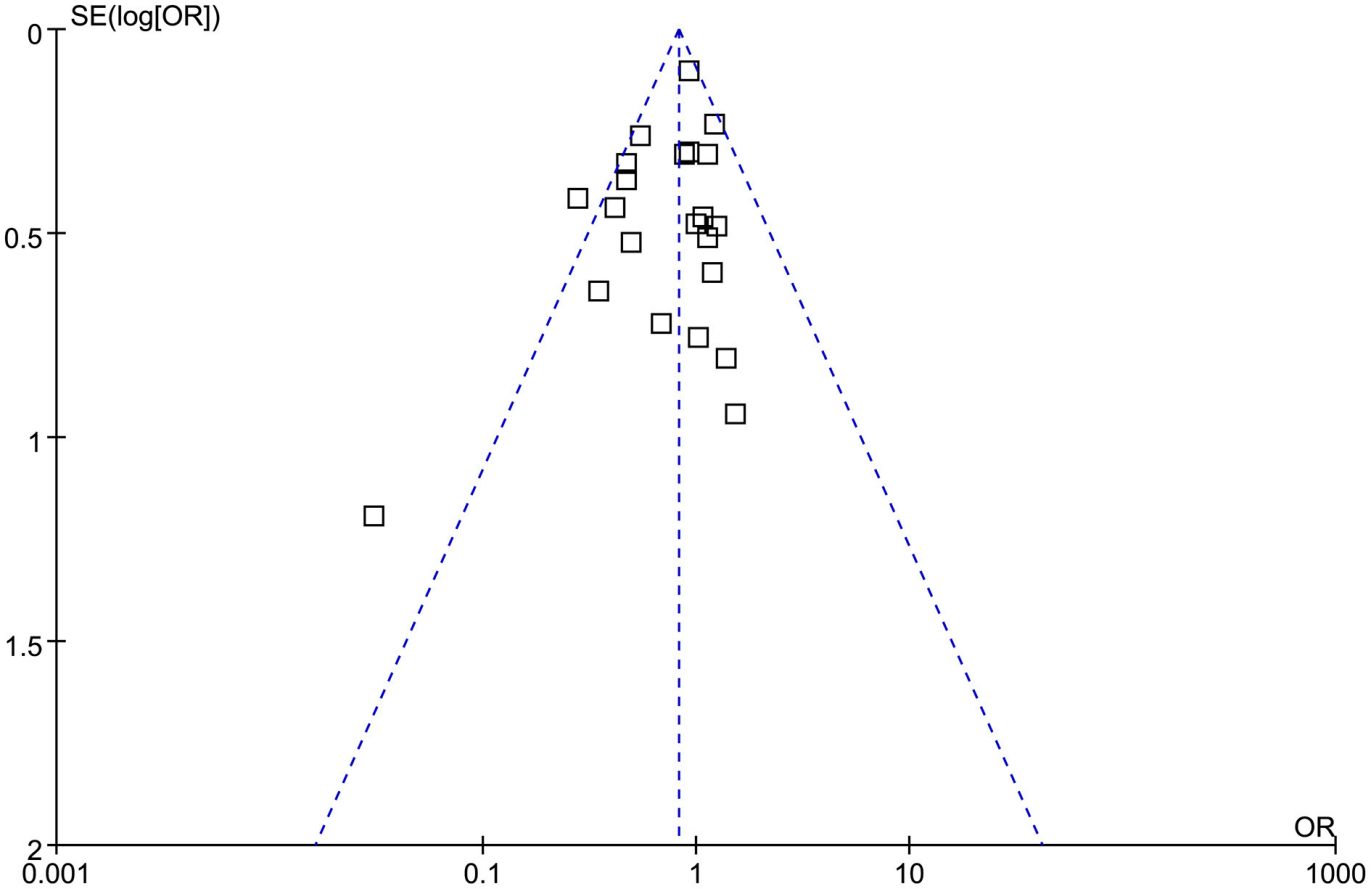
Funnel plots for the meta-analysis comparing the efficacy of RIPC and control on AKI after cardiac surgery.

The funnel plots were symmetrical, suggesting low-risk of publication bias (Figure 5). Egger's regression tests showed similar results (*p* = 0.28).

## Discussion

In this updated meta-analysis, we pooled the results of 22 RCTs that involved 5,389 patients who received cardiac surgery and found that RIPC significantly reduced the incidence of postoperative AKI in these patients. Sensitivity analyses showed consistent results in studies with on-pump surgery only, in studies of patients with normal renal function at baseline, and in studies evaluating the acute efficacy of RIPC. In addition, results of meta-regression and subgroup analyses indicated that characteristics, including study design, country, age, gender, diabetic status, surgery type, use of propofol or volatile anesthetics, cross-clamp time, RIPC protocol, definition of AKI, and sample size of the RCT did not significantly impact the efficacy of RIPC on postoperative AKI. Moreover, stratified analysis showed that RIPC significantly reduced the risk of mild-to-moderate AKI that did not require RRT but did not significantly reduce the risk of severe AKI that required RRT in patients after cardiac surgery. To sum up, these results suggest that RIPC is effective for preventing AKI after cardiac surgery, which seems to be mainly driven by the reduced mild-to-moderate AKI events that did not require RRT. Further efforts are needed to determine the influences of patient characteristics, procedure, perioperative drugs, and RIPC protocol on AKI outcome.

Some meta-analyses have been previously published evaluating the use of RIPC for preventing AKI after cardiac surgery (32–35). Compared with these studies, our updated meta-analysis has the following strengths. First, we included the most recent RCTs with more than 5,000 patients, which is a much larger sample population than was included in the previous meta-analyses. The relatively large number of available RCTs and sample size of this meta-analysis may overcome the potential statistical inadequacy of previously published individual RCTs and meta-analyses. Secondly, some RCTs (41, 42) evaluating the efficacy of combined remote ischemic pre- and post-conditioning, rather than RIPC alone, on AKI were included in previous meta-analyses (34, 35), which may have confounded the results. In our meta-analysis, only studies that compared RIPC and control on AKI after cardiac surgery were included, which therefore reflects the potential benefits of RIPC only. Finally, the relative larger number of available RCTs enables us to perform comprehensive meta-regression and subgroup analyses of the influences of study characteristics on AKI outcome. This is important because these factors were rarely considered in previous meta-analyses, probably due to the limited RCTs available. In this study, we synthesized the current evidence from RCTs and showed that RIPC is effective for preventing AKI after cardiac surgery. Besides the potential preventative efficacy on AKI, previous studies have shown that RIPC is also effective to attenuate perioperative myocardial injury (43) and reduce the risk of new onset atrial fibrillation (44) in patients undergoing cardiac surgery and may reduce mortality in patients receiving volatile inhalational agent anesthesia (34). Taken together, these findings support the benefits of RIPC use for patients undergoing cardiac surgeries.

Moderate heterogeneity was detected among the included studies in our meta-analysis. Although we aimed to evaluate the potential contribution of study characteristics on AKI outcome, results of meta-regression and subgroup analyses did not reveal any significant relationship between these factors and the efficacy of RIPC on postoperative AKI. It has been suggested by previous meta-analyses that patient age, complexity of the surgical procedure, and use of propofol may significantly modify the efficacy of RIPC on postoperative AKI (32, 35). However, by including more eligible RCTs, we found that these factors played no significant role on the outcome of AKI following RIPC. In fact, some recently published experimental studies suggest that the potential interaction between these factors, such as propofol, with organ protective efficacy of RIPC may be more complicated than expected. A recent study in a rat model found that the timing of propofol administration could affect RIPC-induced cardioprotection (45), and that different doses of propofol used in the studies may have also affected the results. Future studies are needed to determine the influences of patient characteristics, procedure, perioperative drugs, and RIPC protocol on the efficacy of RIPC on AKI prevention.

The potential mechanisms underlying the preventative role of RIPC on AKI after cardiac surgery are likely multifactorial (8, 9). Generally, RIPC is considered to mediate organ protection via regulating both the sympathetic and parasympathetic components of the autonomic nervous system (46–48). Ischemia has been associated with activation of sensory (afferent) C-fiber neurons, which trigger the vagus nerve of a distant organ, such as the kidney, consequently exerting protective effects (49). Experimental studies showed that RIPC was associated with the release of various humoral factors, which confers anti-inflammatory and anti-oxidative efficacies (50), two predominant pathogenic mechanisms in AKI. However, the exact mechanisms and key molecular pathways involved in the potential renoprotective efficacy of RIPC remain to be determined.

Our study has some limitations. First, the definitions of AKI varied across the included studies. Although the difference between subgroups was not significantly different, our subgroup analyses suggest that RIPC reduced AKI after cardiac surgery in studies that applied the AKIN definition but not in those that used the RIFLE or KDIGO definition. Since the optimal definition of AKI has yet to be established (51), we could not exclude the possibility that difference in the definition of AKI may affect the outcome of the meta-analysis. Secondly, no access to individual patient data was obtained. Therefore, subgroup analyses were based on study-level data, and results of the subgroup analyses should be interpreted with caution. Future large-scale RCTs or meta-analysis based on individual patient data are needed to validate the results. Moreover, the sample sizes of the included RCTs varied significantly. The two RCTs with largest sample sizes comprised over half of the included patients of the meta-analysis, which may primarily contribute to the results of overall meta-analysis and therefore comprise the importance of the meta-analysis. Besides, variations in the details of protocols for RIPC may affect its influence on AKI. We have performed subgroup analysis to evaluate the potential difference of RIPC performed in upper and lower limbs. However, influences of other details of RIPC, such as number of cycles, pressure applied and durations, and more importantly, the combinations of these factors could not be evaluated in the current meta-analysis. Finally, we did not perform stratified analyses according to the degree of AKI since data regarding the severity of AKI were rare in the included studies. Future studies are warranted in this regard.

In conclusion, results of this updated meta-analysis suggest that RIPC is effective for preventing AKI after cardiac surgery, which seems to be mainly driven by the reduced mild-to-moderate AKI events that did not require RRT. More studies are warranted to determine the influence of patient characteristics, procedure, perioperative drugs, and RIPC protocol on AKI outcome.

## Data Availability Statement

The raw data supporting the conclusions of this article will be made available by the authors, without undue reservation.

## Author Contributions

ZL and YZ contributed to the conception and design of the study. ZL, YZ, ML, and GZ performed the statistical analysis. ZL, YZ, DL, and RS wrote the first draft of the manuscript. XL wrote sections of the manuscript. All authors contributed to manuscript revision and read and approved the submitted version.

## Conflict of Interest

The authors declare that the research was conducted in the absence of any commercial or financial relationships that could be construed as a potential conflict of interest.
